# Patients with Acromegaly Presenting with Colon Cancer: A Case Series

**DOI:** 10.1155/2016/5156295

**Published:** 2016-11-29

**Authors:** Murray B. Gordon, Samer Nakhle, William H. Ludlam

**Affiliations:** ^1^Allegheny Neuroendocrinology Center, Departments of Medicine and Neurosurgery, Allegheny General Hospital, 320 East North Avenue, Pittsburgh, PA 15212, USA; ^2^Palm Research Center, 9280 West Sunset Road, Suite 306, Las Vegas, NV 89148, USA; ^3^Novartis Pharmaceuticals, 1 Health Plaza, East Hanover, NJ 07936, USA

## Abstract

*Introduction*. Frequent colonoscopy screenings are critical for early diagnosis of colon cancer in patients with acromegaly.* Case Presentations*. We performed a retrospective analysis of the incidental diagnoses of colon cancer from the ACCESS trial (ClinicalTrials.gov identifier: NCT01995734). Colon cancer was identified in 2 patients (4.5%). Case  1 patient was a 36-year-old male with acromegaly who underwent transsphenoidal surgery to remove the pituitary adenoma. After surgery, the patient underwent routine colonoscopy screening, which revealed a 40 mm tubular adenoma in the descending colon. A T1N1a carcinoma was surgically removed, and 1 of 22 lymph nodes was positive for metastatic disease, leading to a diagnosis of stage 3 colon cancer. Case  2 patient was a 50-year-old male with acromegaly who underwent transsphenoidal surgery to remove a 2 cm pituitary adenoma. The patient reported severe cramping and lower abdominal pain, and an invasive 8.1 cm^3^ grade 2 adenocarcinoma with signet rings was identified in the ascending colon and removed. Of the 37 lymph nodes, 34 were positive for the presence of tumor cells, and stage 3c colon cancer was confirmed.* Conclusion.* Current guidelines for colonoscopy screening at the time of diagnosis of acromegaly and at appropriate follow-up intervals should be followed.

## 1. Introduction

Acromegaly is a disease most often caused by benign somatotrophic pituitary adenomas that lead to elevated secretion of growth hormone (GH), which stimulates increased expression of insulin-like growth factor 1 (IGF-1) [1]. A persistent increase in GH and IGF-1 leads to known acromegaly-associated comorbidities, including congestive heart failure, arthritis, and impaired glucose tolerance [1]. In some studies, colon polyps or cancer have also been reported to occur more frequently in patients with acromegaly than in the general population [2]. However, data from other studies have not supported the association between acromegaly and colon cancer [2, 3]. Here, we present case studies of 2 patients who were diagnosed with colon cancer from a small cohort of patients with acromegaly.

## 2. Case Presentations

We performed a retrospective analysis of the incidental diagnoses of colon cancer in the ACCESS trial (ClinicalTrials.gov identifier: NCT01995734), an open-label, multicenter study that allowed for expanded access to pasireotide long-acting while regulatory approval was being pursued. To be included in the cohort, patients with acromegaly must have undergone surgery to remove the pituitary tumor (unless they were not eligible for surgery or refused surgery) and had uncontrolled acromegaly, as defined by IGF-1 levels greater than the upper limit of normal and random GH greater than 1 ng/mL. Patients were excluded if they had been diagnosed with active malignant disease within the last 5 years (with the possible exception of basal cell carcinoma or carcinoma in situ of the cervix). Colon cancer was diagnosed using colonoscopy, and histological examination of tumor resections was used for cancer staging.

The cohort was composed of 44 patients (female, 56.8%). The mean age was 45.5 years, and the mean body mass index was 32.9 kg/m^2^. Impaired fasting glucose and type 2 diabetes mellitus were observed in 1 (2.3%) and 9 (20.5%) patients, respectively. Two men (4.5%) were incidentally diagnosed with stage 3 colon cancer soon after they entered the study (after 1 week for 1 patient and after 5 months for the other patient); however, the cancer-related events were considered to be unrelated to treatment with the study drug.

**Figure 1:**
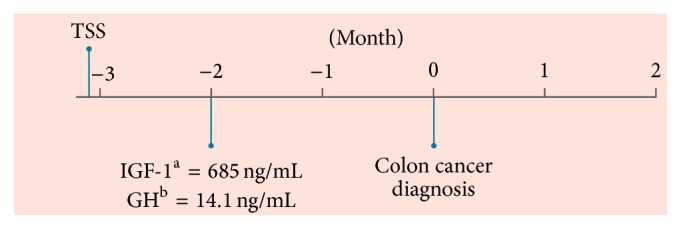
Patient timeline from removal of pituitary adenoma to colon cancer diagnosis. GH, growth hormone; IGF-1, insulin-like growth factor 1; TSS, transsphenoidal surgery. ^a^Reference range, 109–329 ng/mL. ^b^Reference range, ≤3 ng/mL.

Case  1 patient was a 36-year-old white male with a reported family history of colon cancer (grandmother) who presented with acromegaly, weight loss, and skin tags. The patient had not been screened by colonoscopy before diagnosis of acromegaly. Three months before case presentation, transsphenoidal surgery was performed with resection of a pituitary adenoma (Figure 1). Approximately 6 weeks after surgery, the patient's IGF-1 level was 685 ng/mL, more than twice the upper limit of normal (reference range, 109–329 ng/mL), and his GH level was 14.1 ng/mL (reference range, ≤3 ng/mL). Magnetic resonance imaging revealed no evidence of pituitary adenoma 3 months after surgery.

The patient enrolled in the study 1 week before case presentation. One week after study enrollment, the patient underwent routine colonoscopy screening, which revealed a 40 mm tubular adenoma in the descending colon. A T1N1a carcinoma was surgically removed 2 weeks later; 1 of 22 lymph nodes was positive for metastatic disease. The patient was diagnosed with stage 3 colon cancer and started oxaliplatin every 3 weeks (targeting 10 treatments) and capecitabine 4 g once daily. The patient reported cold sensitivity, some nausea, soft stools (resolved), weight loss, metallic mouth, depression, and anxiety associated with chemotherapy treatment. The colon tumor was subsequently resected, and the patient is now in remission without evidence of tumor recurrence.

**Figure 2:**
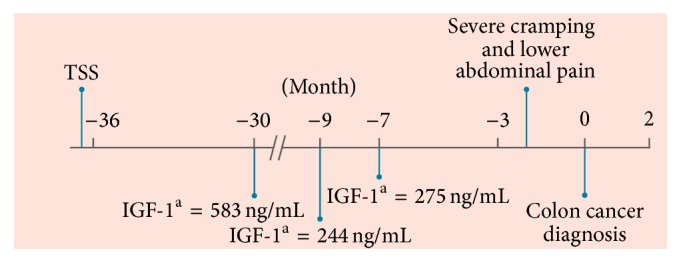
Patient timeline from removal of pituitary adenoma to colon cancer diagnosis. IGF-1, insulin-like growth factor 1; TSS, transsphenoidal surgery. ^a^Reference range, 70–205 ng/mL.

Case  2 patient was a 50-year-old male who originally presented with headache, hypogonadism, and fatigue. Initial colonoscopy (performed 3 years before the diagnosis of acromegaly) was negative. Magnetic resonance imaging showed a 2 cm pituitary adenoma that was removed with transsphenoidal surgery. The patient's IGF-1 levels were uncontrolled after surgery and ranged from 244 to 583 ng/mL (reference range, 70–205 ng/mL) during 16 months of lanreotide Autogel treatment (Figure 2). The patient switched to pasireotide long-acting at the beginning of the trial and continued to receive pasireotide.

Six months later, the patient reported severe cramping and lower abdominal pain, and a colonoscopy was performed. Two polyps were revealed, and an invasive 8.1 cm^3^ grade 2 adenocarcinoma with signet rings was removed from the ascending colon. Of the 37 lymph nodes, 34 were positive for the presence of tumor cells. Histology confirmed stage 3c colon cancer, and the patient started treatment with capecitabine and oxaliplatin. The patient subsequently died in hospice care because of metastatic colon cancer. The death was not considered related to pasireotide treatment.

## 3. Discussion

These case studies detail the identification of advanced-stage colon cancer in 2 patients (4.5%) from a cohort of patients who were being treated for uncontrolled acromegaly. The incidence of colon cancer in this cohort is comparable to and within the range of other reported epidemiological findings of colon cancer in acromegaly (1.1%–20.0%) [4]. It should be noted that the identification of colon cancer was not considered to be related to the study drug, which is not surprising given that the 2 patients had only been in the study for 1 week in case  1 and 5 months in case  2, and colon cancer is associated with various stages that occur over many years. Because many patients with uncontrolled acromegaly are undiagnosed for years, it is possible that persistently elevated GH and IGF-1 levels could be a factor in the relatively high incidence of colon cancer in this cohort compared with the general population [5]. Therefore, biochemical control of GH and IGF-1 levels may reduce the risk of colon cancer in patients with acromegaly. There are currently no prospective interventional studies demonstrating to what extent therapies that reduce GH and IGF-1 levels affect the incidence and prognosis of colon cancer in acromegaly.

The extent of the association between colon cancer risk and acromegaly has been unclear. In a preclinical model, local increases in colon GH generated a tumor microenvironment that was permissive for neoplastic colon growth in mice [6]. In addition, in a meta-analysis that included 9 controlled studies of 701 patients with acromegaly and 1573 controls, there was a higher risk of colon cancer in patients with acromegaly (14/304 [4.6%]) than in control patients (8/627 [1.2%]) [2]. In contrast, a study by Renehan et al. found no difference in the prevalence of colon cancer or colonic polyps between patients with acromegaly using colonoscopy and control patients without acromegaly using autopsy examinations [7]. However, autopsy examinations may have detected a higher rate of adenomas/carcinomas in the general population because the examinations could be more extensive.

Other factors may contribute to the increased risk of colon cancer in patients with acromegaly. Hyperplastic polyps and carcinoma have been associated with higher levels of serum GH [8], although higher IGF-1 levels are also associated with increased risks [9, 10]. In addition, the risk of colonic lesions is 2.4- to 5.8-fold higher in patients with impaired glucose regulation (impaired fasting glucose, impaired glucose tolerance, or diabetes) than in those with normal glucose tolerance [11]. This effect may be mediated by hyperinsulinemia, because the risk of developing adenomatous polyps was 14.8 times higher in patients with fasting insulin levels in the highest tertile than in those with levels in the lowest tertile [11]. Additionally, genetic factors have also been associated with more frequent incidences of colorectal cancer and may increase the risk of colorectal cancer in patients with acromegaly [12].

Acromegaly treatment guidelines from the American Association of Clinical Endocrinologists (AACE) and the Endocrine Society suggest screening for colon neoplasia with colonoscopy at the time of diagnosis of acromegaly [13, 14]. Furthermore, AACE guidelines suggest that follow-up colonoscopy be performed at time intervals “appropriate for patients at higher-than-average risk for colon cancer” [13], whereas the Endocrine Society guidelines suggest screening every 5 years for patients with elevated IGF-1 levels or polyps and every 10 years for patients with normalized IGF-1 levels and no polyps [14]. In contrast, guidelines from the British Society of Gastroenterology recommend screening every 3 years for patients with adenoma(s) at initial colonoscopy and/or elevated IGF-1, while those from the Acromegaly Consensus Group recommend screening every 3 to 5 years depending on the number and size of the adenomas [4]. These guidelines provide 3 points of consideration. First, colonoscopy was shown to be superior to fecal occult blood testing in identifying adenomas and cancer and is the suggested method of screening for colon neoplasms [15]. Second, colonoscopies should be performed at diagnosis, as evidenced in a study that showed that up to 19.3% of patients with acromegaly who were aged <40 years compared with 4.4% of controls had colonic neoplasia at diagnosis [10]. Third, follow-up colonoscopies should occur, although the frequency has not been firmly established. In this study, there was no evidence of colon cancer in 1 patient at his previous colonoscopy (3 years before the diagnosis of acromegaly and 5 years before the diagnosis of colon cancer). In a retrospective study of patients with acromegaly who were screened by colonoscopy at a mean interval of approximately every 4 years, new polyps were identified in roughly one-third of patients at each screening [16]. Additionally, in this study, a patient with a family history of colon cancer was reported. A cohort analysis previously showed a potential association between increased risk of colon cancer and acromegaly in male patients with a family history of colon cancer [17]. A joint guideline from the American Cancer Society, US Multi-Society Task Force on Colorectal Cancer, and American College of Radiology recommends that patients with a family history of colon cancer should have a colonoscopy at an earlier age (i.e., before the generally recommended age of 50 years) and more frequently than individuals at average risk [18]. Therefore, based on this case series and in conjunction with a literature review, we suggest an evidence-based guideline of follow-up screening with colonoscopy at relatively shorter intervals (e.g., every 3 years) in patients with acromegaly who are ≤50 years of age, particularly in cases with a positive family history of colon cancer.

## 4. Conclusion

The diagnoses of colon cancer in this cohort of patients with acromegaly suggests that physicians should perform a routine colonoscopy screening at the time of diagnosis of acromegaly and at appropriate follow-up intervals after the diagnosis. Furthermore, control of GH and IGF-1 levels should be paramount to mitigate comorbidities such as colon cancer.
